# Systematic review and meta-analysis: Evaluating the influence of intrahepatic cholestasis of pregnancy on obstetric and neonatal outcomes

**DOI:** 10.1371/journal.pone.0304604

**Published:** 2024-06-04

**Authors:** Xuexia Huang, Huifeng Gu, Pinghua Shen, Xiaoxing Zhang, Anping Fei

**Affiliations:** Obstetrical Department, Huzhou Maternal and Child Health Hospital, WuXing District, Huzhou City, Zhejiang Province, China; Kermanshah University of Medical Sciences, ISLAMIC REPUBLIC OF IRAN

## Abstract

**Background:**

Intrahepatic cholestasis of pregnancy (ICP) is a serious liver conditions that negatively impacts obstetric and neonatal outcomes. Elevated levels of bile acid, particularly glycine conjugate, may compromise blood flow and cause functional hypoxia-ischemia.

**Aims:**

This meta-analysis aims to assess the association between ICP and key pregnancy outcomes including emergency caesarian sections (C-sections), preeclampsia, hemorrhage, preterm birth, small for gestational age, admission rate to neonatal intensive care union (NICU), gestational age, and stillbirth.

**Materials and methods:**

Literature search across five databases (PubMed, Embase, Web of Science) was done to detect relevant studies published up until June 2023. Meta-analysis of the identified studies was done using a random-effects model, and the results presented as Odds ratio (OR).

**Results:**

A literature search identified 662 studies. Of them, 21 met the inclusion criteria. There was a significant association between ICP and odds of C-section (OR: 1.42, p <0.001), preeclampsia (OR: 2.64, p <0.001), NICU admission (OR: 2.1, p <0.001), and pre-term birth (OR: 2.64, p <0.001). ICP was not associated with postpartum hemmorhage (OR: 1.31, p = 0.13), small for gestational age (OR: 0.87, p = 0.07), stillbirth (OR: 1.49, p = 0.29).

**Conclusions:**

Our results confirm the adverse effects of ICP on co-existing pregnancy complications, obstetric and neonatal outcomes. ICP in associated with severe complications including increased rates of preeclampsia, emergency C-sections, preterm births, l gestational periods and higher rates of NICU admissions. These results may assist healthcare professionals in formulating comprehensive care guidelines for expectant mothers and newborns.

## Introduction

Intrahepatic cholestasis of pregnancy (ICP) typically presents in the second a third trimesters and may be caused by various factors such as hormones, inflammation, immune responses, and genetic factors, as well as pre-existing hepatobiliary conditions [1–3]. The reported incidence of ICP varies widely, ranging from less than 1% to 27.6%. Higher rates of ICP are associated with certain geographical regions and specific risk factors such as chronic hepatitis C, personal or family history of intrahepatic cholestasis, and advanced maternal age [2, 4–6].

ICP can lead to poor obstetric and neonatal outcomes through several mechanisms. Elevated levels of bile acids in the maternal bloodstream can impair placental function, reducing the supply of oxygen and nutrients to the fetus and potentially causing fetal distress and growth restriction [7–11]. Stimulating effect of bile acids on uterine contractions may lead to increased risk of preterm labor and delivery [9, 10, 12–14]. Severe cases of ICP may also result in meconium staining, where the fetus passes fecal material into the amniotic fluid, which can lead to aspiration and respiratory complications [4, 7, 8, 15–17]. Altered placental function and reduced oxygen supply may contribute to fetal distress, characterized by the changes in heart rate patterns and oxygen deprivation during labor and delivery [9, 10, 18]. There are also reports of the link between ICP and an increased risk of stillbirth that is possibly related to the impact of elevated bile acids on placental function and fetal well-being [7, 8, 13].

Clinical signs of ICP manifest in generalized itching, primarily in the palms and soles [1, 5, 6], nausea and decreased appetite [1, 5, 10, 19]. Diagnosis of ICP is done based on detection of pruritus along with raised levels of total serum bile acid and/or aminotransferases, while ruling out other potential confounding diseases [1, 2, 6]. ICP has been associated with adverse fetal and obstetric outcomes, such as neonatal respiratory distress syndrome, high rates of stillbirths, preterm delivery (spontaneous and induced), and high rates of unplanned C-sections due to complications [1, 5, 6, 10, 18]. Moreover, the reported incidence of stillbirth attributed to ICP is approximately 0.7–0.9% (Atabey et al., 2007; Smith & Rood, 2020). Although some risks appear to increase with higher bile acid levels and advancing gestational age, the data regarding obstetric and fetal risks is inconsistent [3, 5, 6].

Several studies have attempted to evaluate the association between ICP and obstetric and neonatal outcomes [7, 8, 13]. While some studies have reported a substantial worsening of obstetric outcomes in patients with ICP, such as higher incidence of unplanned/emergency C-sections, preeclampsia, postpartum Hemorrhage, others have found no such association [1, 5, 6, 14, 20, 21]. There is also no consensus in terms of the association between ICP and neonatal outcomes such as gestational age, preterm birth, small for gestational age, NICU admission, and stillbirth [5, 14, 15, 20–25]. Currently, no attempts were made to summarize the existiong data that evaluates the association between ICP and obstetric and neonatal outcomes.

This study aims to investigate the associations between severe ICP and co-existing pregnancy complications, obstetric and neonatal outcomes such as incidence of preeclampsia, unplanned/emergency C-sections, postpartum hemorrhage, duration of gestational age, preterm birth, small for gestational age, NICU admission, and stillbirth. Our results may provide valuable guidance for clinicians and nursing personnel in selecting appropriate strategies and effectively managing ICP patients.

## Methods

The Preferred Reporting Items for Systematic Reviews and Meta-Analyses (PRISMA) were followed [26]. The study is registered at PROSPERO, with the No. CRD42023432927.

### Search strategy

A comprehensive literature search across electronic databases, including PubMed, Embase, Web of Science was done for papers, published from inception until June 2023, exploring ICP and its impact on fetal growth and development. A detailed search strategy for all the included databases has been provided in S1 File. The search terms used included "intrahepatic cholestasis of pregnancy," "ICP," "itching of the skin," "jaundice," "abnormal liver function," "fetal growth," "complications," "NICU admissions," "gestational age," "stillbirth," "APGAR score," "premature delivery," "neonatal morbidity," "bile acid levels," "cholic acid glycine conjugate," and "placental villi." Rayyan software was used to screen and eliminate duplicate studies [27]. Additionally, bibliography of relevant articles were screened manually for any additional eligible studies.

Inclusion Criteria (as per the PECOS criteria):

1. Participants (P): Pregnant individuals diagnosed with intrahepatic cholestasis of pregnancy (ICP).
2. Exposure (E): Studies investigating the impact of ICP on obstetric and neonatal health outcomes.
3. Comparators (C): Studies comparing outcomes between ICP and non-ICP groups.
4. Outcomes (O): Co-existing pregnancy complications: Preeclampsia. Obstetric outcomes: Unplanned/emergency C-sections, postpartum hemorrhage, gestational age, preterm birth, small for gestational age. Neonatal outcomes: Stillbirth and NICU admissions.
5. Study Design (S): Clinical trials, observational studies, or systematic reviews.
6. Language (L): Studies written in the English language.
7. Population (P): Human participants.

The inclusion criteria encompass studies that focus on the impact of ICP on obstetric and neonatal outcomes, comparing these outcomes between ICP and non-ICP groups. The criteria include various relevant outcomes, study designs, language requirements, and the specific population of interest.

Exclusion criteria:

1. Studies not directly related to intrahepatic cholestasis of pregnancy or its associated factors.
2. Articles focused on other liver diseases or conditions not specifically addressing ICP.
3. Research studies with a small sample size (e.g., case reports, case series) that may lack statistical power or generalizability.
4. Animal studies, in vitro experiments, or studies conducted on non-human subjects.
5. Articles published in languages other than English and without a reliable English translation available.

Two reviewers independently conducted literature search and assessment of the identified studies to determine the eligibility for inclusion in the review and meta-analysis. Each study was evaluated based on predetermined eligibility criteria. This approach minimizes bias and enhances the accuracy of study selection.

A third reviewer was consulted in all cases of disagreement regarding the final selection of studies to ensured a comprehensive and impartial selection of studies for inclusion in the meta-analysis.

### Quality assessment

Potential risk of bias in the cohort trials was evaluated using the ROBINS-I tool [28]. The methodological quality of the included studies was independently evaluated by two reviewers, and any discrepancies were resolved through consultation with a third reviewer acting as an arbitrator. This rigorous evaluation process ensured a comprehensive and unbiased assessment of the studies’ methodological strengths and limitations.

### Data extraction

A meticulous data extraction was performed to gather relevant information from the included studies. Key data points, such as the study type, groups involved, sample size, and average maternal age, were recorded. Data on obstetric outcomes, including rates of unplanned/emergency C-sections, preeclampsia, postpartum Hemorrhage, gestational age, preterm birth, small for gestational age, NICU admission, and stillbirth were collected. We defined lower gestational age as pregnancies that had progressed less than 37 weeks of gestation [29]. We extracted information related to the evaluation of risk estimates and the specific parameters that were collected and analyzed in the included studies. This comprehensive data extraction process enabled us to thoroughly examine the association between these variables and obtain valuable insights into their potential impact on maternal and fetal health outcomes.

### Data analysis

Comprehensive Meta-analysis version 3.0 and a random-effects model were used for data analysis [30]. A meta-analysis was conducted to examine the association between ICP and adverse co-existing pregnancy complications, obstetric and neonatal outcomes by pooling descriptive statistics. This analysis provided an overall estimation of the relationship between ICP and the incidence of adverse maternal and neonatal outcomes. To assess the impact of ICP on various adverse fetal outcomes and co-existing pregnancy complications, including rates of unplanned/emergency C-sections, preeclampsia, postpartum hemorrhage, gestational age, preterm birth, small for gestational age, NICU admission, and stillbirth, odds ratios were calculated. We used weighted effect sizes in the form of standard difference in mean to compute length of gestational age. Heterogeneity was evaluated using I^2^ statistics. I^2^ of 0–25% indicated negligible heterogeneity, I^2^ of 25–85% indicated moderate heterogeneity, and I^2^ ≥75% indicated substantial heterogeneity [31]. To assess publication bias, we employed Duval and Tweedy’s trim and fill procedure [32]. All analyses in this study were conducted with a significance level of 5%.

## Results

### Systematic review report

#### General characteristics of included studies

**Fig 1:**
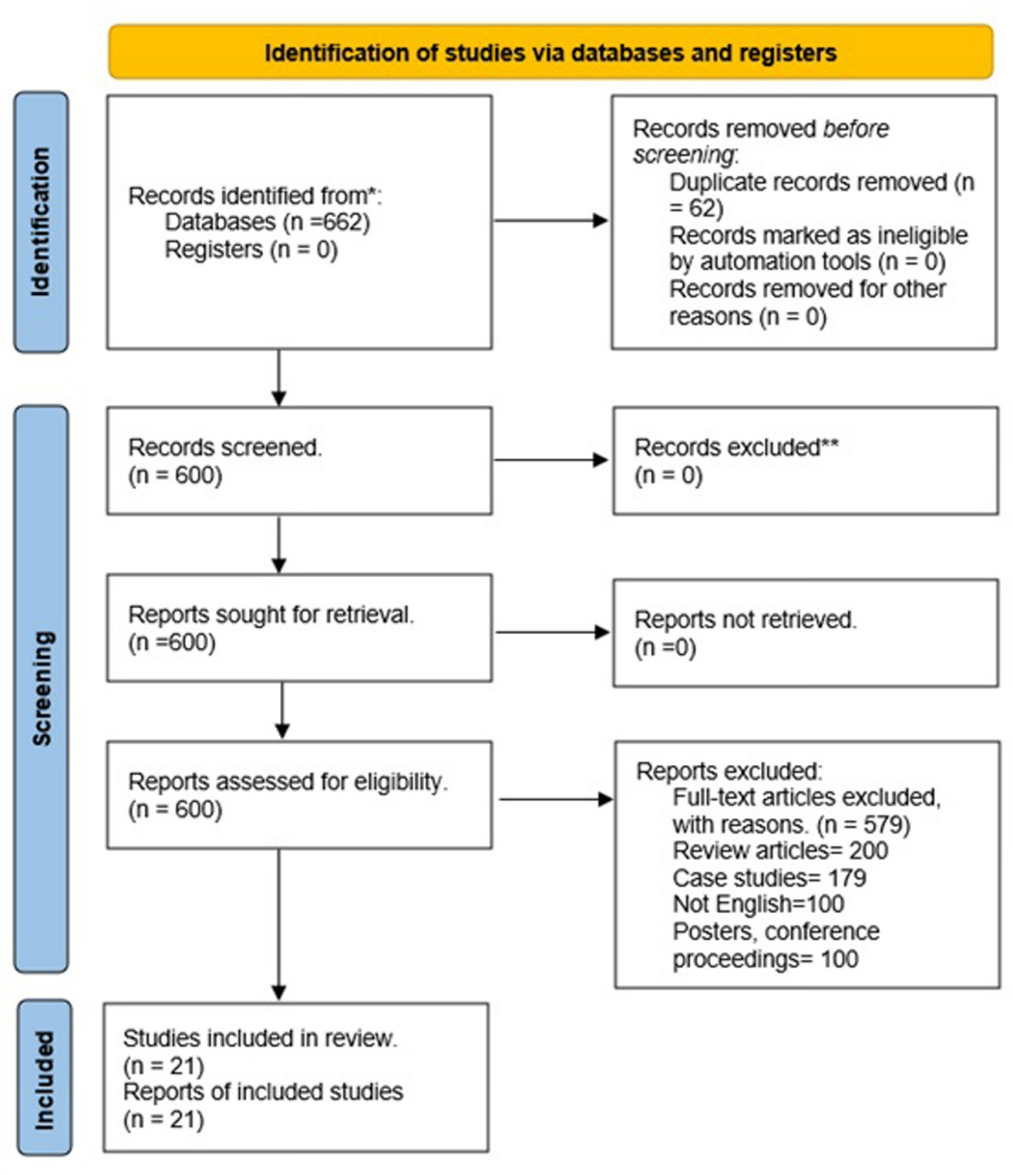
PRISMA flowchart.

Systematic literature search identified 662 studies. Additional eight studies were identified in the bibliography section of the retrieved papers. A total of 21 studies met the eligibility criteria and were included in the analysis (Fig 1), with a heterogeneity in terms of study designs. Eleven studies were prospective cohort studies [8, 9, 13, 21–23, 25, 33–36] and ten were retrospective cohort studies [7, 11, 12, 14–17, 20, 24, 37]. The extracted data from all studies are presented in detail in Table 1.

**Table 1:** Details of included studies.

Study	Country	Type of Study	Number of pregnant mothers		Average Maternal age (years)	Obstetric outcomes			Neonatal outcomes				
			ICP	No-ICP		Postpartum hemorrhage	C-Section	Preeclampsia*	Gestational age Weeks	NICU admission	Stillbirth	Preterm delivery	Small for gestational age
Chen et al. (2023)	China	Retrospective cohort study	688	38556	ICP: 29 ± 4 No-ICP: 29 ± 4	ICP:0.15% Non-ICP:0.28%	ICP: 51.31% Non-ICP: 31.6%	ICP: 4.94% Non-ICP: 1.56%	ICP: 38.5 Non-ICP: 38.8	NR	ICP: 0.15% Non-ICP: 0.44%	ICP: 18.6% Non-ICP: 4.87%	NR
Herrera et al. (2018)	United States of America	Retrospective cohort study	487	298	ICP: 29 ± 5 Non-ICP: 29 ± 5	NR	ICP: 32% Non-ICP: 29.9%	NR	ICP: 36.5 Non-ICP: 37.3	NR	ICP: 2.9% Non-ICP: 0.3%	ICP: 9.4% Non-ICP: 6.1%	ICP: 2.19% Non-ICP: 1.45%
Martineau et al. (2014)	United Kingdom	Retrospective cohort study	143	57581	ICP: 30.8 ± 4 Non-ICP: 30.6 ± 4		ICP: 33% Non-ICP: 29%	NR	NR	NR	ICP:2.1% Non-ICP:0.3%	NR	NR
Wang et al. (2019)	China	Retrospective cohort study	40	40	ICP: 29.5 ± 3 Non-ICP: 29.5 ± 3	NR	NR	NR:	ICP: 34.1 Non-ICP: 38.9	NR	NR	NR	NR
Luo et al. (2021)	China	Retrospective cohort study	114	3725	ICP: 30 No-ICP: 30	ICP: 0 Non-ICP: 0.7%	ICP:35.09% Non-ICP: 21.93%	ICP: 10.5% Non-ICP: 0.7%	ICP:39 Non-ICP:39.7	NR	ICP:0 Non-ICP:0	ICP:6.14% Non-ICP: 0.18%	NR
Geenes et al. (2014)	United Kingdom	Prospective cohort study	669	2205	ICP: 29.6 Non-ICP: 29	NR	ICP: 25% Non-ICP: 23%	ICP: 6.6% Non-ICP: NR	ICP:37.5 Non-ICP:39.6	ICP:12% Non-ICP: 5.6	ICP:1.5% Non-ICP:0.5%	ICP: 25% Non-ICP: 6.5%	NR
Wu et al. (2022)	China	Retrospective cohort study	1516	38226	ICP: 31.2 Non-ICP: 31.13	NR	ICP: 56.14% Non-ICP: 39.92%	NR	NR	ICP:27.41% Non-ICP: 15.13%	ICP:0.77% Non-ICP: 0.71%	ICP: 22.4% Non-ICP:9.26%	ICP: 4.25% Non-ICP: 4.71%
Al-Obaidly et al. (2022)	Qatar	Retrospective cohort study	263	31230	ICP: 35 Non-ICP: 35	ICP: 5.7% Non-ICP:5.2%	ICP: 27% Non-ICP: 31.3%	ICP:1.5% Non-ICP:1.6%	ICP:32 Non-ICP:36	ICP:15.3% Non-ICP: 11.8%	ICP:0.4% Non-ICP: 0.7%	ICP: 1.4% Non-ICP: 1.4%	NR
Arthuis et al. (2020)	France	Prospective cohort study	140	560	ICP: 29 Non-ICP: 29	ICP:20.7% Non-ICP: 15.35%	ICP: 13.6% Non-ICP: 10.9%	NR	ICP: 38 Non-ICP: 40	ICP:2.9% Non-ICP:0.4%	ICP:0 Non-ICP: 0	ICP: 15.7% Non-ICP: 4.8%	ICP: 5.7% Non-ICP: 3.2%
Begum et al. (2023)	India	Prospective cohort study	60	60	ICP: 27.63 Non-ICP: 26.73	NR	ICP: 58.3% Non-ICP: 35%	NR	ICP: 37.8 Non-ICP: 38.07	ICP:21.6% Non-ICP: 5%	ICP:0 Non-ICP: 0	ICP: 21.7% Non-ICP:10%	NR
Wikström Shemer et al. (2013)	Sweden	Prospective cohort study	5477	1208191	NR	NR	ICP: 19.6% Non-ICP: 15.6%	ICP: 6.7% Non-ICP: 2.7%	NR	NR	ICP:0.3 Non-ICP: 0.3	ICP: 0.5% Non-ICP: 0.7%	ICP: 1.1% Non-ICP: 2.3%
Turunen et al. (2010)	Finland	Retrospective cohort study	687	1374	ICP: 27.5 Non-ICP: 27.2	NR	ICP: 14.8% Non-ICP: 10.6%	NR	NR	NR	ICP:1.2% Non-ICP: 0.7%	ICP:44% Non-ICP: 12%	NR
Padmaja et al. (2010)	India	Retrospective case-control study	45	90	ICP: 28.7 Non-ICP: 28.5	ICP: 0 Non-ICP: 0	ICP: 93.3% Non-ICP: 76.7%	NR	NR	ICP: 15.6% Non-ICP: 15.6%	ICP:1% Non-ICP: 4	ICP: 24.4% Non-ICP: 15.6%	NR
Heinonen and Kirkinen (1999)	Finland	Prospective cohort study	91	16818	NR	NR	ICP: 25.3% Non-ICP: 15.8%	NR	NR	NR	ICP:0 Non-ICP: 0.4%	ICP: 14.3% Non-ICP: 5.5%	ICP: 7.7% Non-ICP: 8.5%
Rioseco et al. (1994)	United States of America	Retrospective case-control study	320	320	NR	NR	ICP:25.9% Non-ICP:16.9%	ICP:6.3% Non-ICP:4.7%	NR	NR	ICP:0.012% Non-ICP:0.009%	ICP: 19.3% Non-ICP: 6.8%	ICP: 6.3% Non-ICP: 4.4%
Kawakita et al. (2015)	United States of America	Retrospective cohort study	26	152	ICP: 30.2Y Non-ICP: 29.7Y	ICP: 12.7% Non-ICP: 9.9%	ICP: 40% Non-ICP:38.2%	ICP: 7.3% Non-ICP: 7.2%	ICP: 33.6 Non-ICP: 34.2	ICP: 20.4% Non-ICP: 27.3%	ICP: 0 Non-ICP: 0	ICP: 46.2% Non-ICP: 14.5%	NR
Furrer et al. (2016)	Switzerland	Prospective cohort study	345	1725	ICP: 27.5Y Non-ICP: 27.7Y	NR:	ICP: 27.5% Non-ICP: 27.7%	NR	ICP: 37.4 Non-ICP:37.4	ICP: 15.9% Non-ICP: 16.8%	ICP: 0.9% Non-ICP: 3.6%	NR	NR
Liu et al. (2016)	China	Prospective cohort study	129	1793	ICP: 32.4Y No-ICP: 30.7Y	NR	NR	ICP:27.9% No-ICP:12.6%	ICP:35.2 No-ICP:35.6	ICP:50.2% No-ICP:34.9%	NR	ICP:28.7% No-ICP:19.2%	ICP:11.5% No-ICP:9.7%
YOONG et al. (2008)	Unied kingdom	Prospective cohort study	33	158	NR	ICP:14.68% No-ICP:1.38%	ICP: 14% No-ICP: 8%	NR	ICP: 39.08 No-ICP: 38.9	NR	ICP:1.4% No-ICP: 0%	ICP:2% No-ICP:1.38%	NR
Al Shobaili et al. (2011)	Saudi Arabia	Prospective cohort study	76	200	ICP:29.18Y No-ICP:29.86Y	ICP:5.2% No-ICP:4.5%	ICP:11.8% No-ICP:8.5%	NR	ICP:36.63 No-ICP:37.24	ICP:15.7% No-ICP:4.9%	NR	ICP:11.8% No-ICP:9%	NR
Liu et al. (2020)	China	Prospective cohort study	911	94817	ICP: 30.8Y Non-ICP: 30.6Y	ICP: 0.9% Non-ICP: 0.8%	ICP: 0.7% Non-ICP: 0.3%	ICP: 5.5% Non-ICP: 2.4%	ICP:37.32 Non-ICP: 38.49	ICP: 29.5% Non-ICP: 13.5%	ICP: 0.1% Non-ICP: 0.3%	ICP: 0.9% Non-ICP: 1.1%	ICP: 3.5% Non-ICP: 2%

ICP: Intrahepatic cholestasis of pregnancy, NR: Not reported, NICU: Neonatal intensive care unit

* Co-existing complication

#### Participant information

The included studies incorporated data of 12260 female patients from the ICP pool and 1498119 female patients from the control non-ICP pool (Fig 1). The mean age of the participants from the ICP and the non-ICP pool was 29.8 ± 2 years and 29.6 ± 2 years, respectively.

#### Assessment of study quality

The methodological quality of the cohort studies is shown in Table 2. There was a generally low risk of bias across the included studies. However, several studies had missing data and showed signs of selection bias, suggesting possible sources of bias (Table 2).

**Table 2:** Risk of bias as per the ROBINS-I methodological tool (low risk of bias: +, high risk of bias: -, lack of clarity:?).

Study	Confounding bias	Selection bias	Deviation from the intervention	Missing data	Measurement of outcomes	Selective reporting	Classification of the intervention
Chen et al. (2023)	+	+	+	+	?	+	+
Herrera et al. (2018)	+	?	+	+	+	+	+
Martineau et al. (2014)	?	?	?	?	?	?	+
Wang et al. (2019)	+	+	+	-	+	+	+
Luo et al. (2021)	+	+	+	+	+	+	+
Geenes et al. (2014)	+	+	+	+	+	+	+
Wu et al. (2022)	+	+	+	+	?	+	+
Al-Obaidly et al. (2022)	+	?	?	?	?	?	+
Arthuis et al. (2020)	+	+	+	+	+	+	+
Begum et al. (2023)	+	+	+	+	+	+	+
Wikström Shemer et al. (2013)	+	?	+	?	+	+	+
Turunen et al. (2010)	+	?	+	?	+	+	+
Padmaja et al. (2010)	+	+	+	+	+	+	+
Heinonen and Kirkinen (1999)	+	?	+	?	+	+	+
Rioseco et al. (1994)	+	?	+	+	+	+	+
Kawakita et al. (2015)	+	+	?	+	+	+	+
Furrer et al. (2016)	+	+	+	+	+	+	+
Liu et al. (2016)	+	+	+	+	?	+	+
YOONG et al. (2008)	+	?	?	?	?	?	+
Al Shobaili et al. (2011)	+	+	+	+	+	+	+
Liu et al. (2020)	+	+	+	+	+	+	+

#### Meta-analysis report

**Fig 2:**
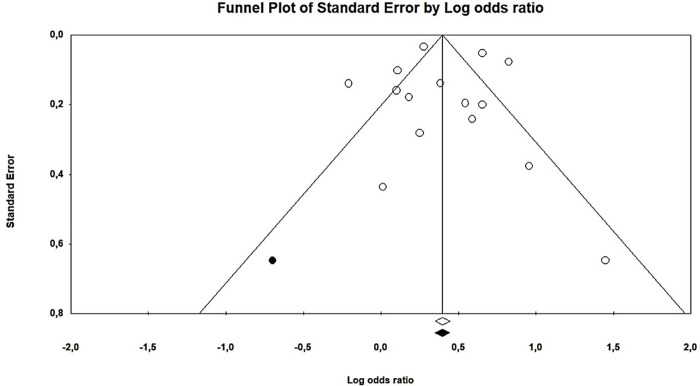
The trim and fill method by Duval and Tweedy provides a visualization of how publication bias can impact study results.

*Publication bias*. Duval and Tweedy’s trim and fill method was used to assess publication bias. This method estimates the number of missing studies on either side of the mean effect of the funnel plot. The results show that one study was missing on the left side of the mean effect. The overall random effect models provided a point estimate and 95% confidence interval (CI) for the studies as 1.42 (95% CI 1.21–1.66). The trim and fill imputed point estimates were calculated as 1.40 (95% CI 1.19–1.64). The results of the publication bias assessment are presented in Fig 2.

### Meta-analysis outcome

#### Obstetric outcomes or Co-existing pregnancy complication

*Association of ICP with unplanned/emergency C-section*. Our analysis of 19 cohort studies indicated that the presence of ICP was significantly associated with higher risks of unplanned/emergency C-sections (odds ratio [OR]: 1.42, 95% confidence interval [CI]: 1.21–1.66, p<0.001). A negligible level of heterogeneity (I^2^ = 84.74%) was observed in this analysis (S1 Fig).

*Association of ICP with preeclampsia*. Our analysis of 9 cohort studies indicated that ICP was significantly associated with higher risks of preeclampsia which is a co-existing pregnancy complication (OR: 2.6, 95% confidence interval [CI]: 2.41–2.89, p<0.001). A moderate level of heterogeneity (I^2^ = 68.3%) was observed in this analysis (S2 Fig).

*Association of ICP with Postpartum hemorrhage*. Our analysis of 8 cohort studies indicated that ICP was significantly associated with higher risks of postpartum hemorrhage (OR: 1.31, 95% confidence interval [CI]: 0.91–1.88, p 0.139). A moderate level of heterogeneity (I^2^ = 13.47%) was observed in this analysis (S3 Fig).

*Association of ICP with lower gestational age*. Our analysis of 14 cohort studies indicated that ICP was significantly associated with lower gestational age (Hedge’s g: -0.50, 95% confidence interval [CI]: 1.15 to 0.02, p = 0.001). No heterogeneity (I^2^ = 9.9%) was observed in this analysis (S4 Fig).

#### Association of ICP with NICU admission

The analysis of 11 cohort studies showed that the presence of ICP was significantly associated with higher risks of NICU admission (OR: 2.10, 95% confidence interval [CI]: 1.95–2.28, p<0.00). A moderate level of heterogeneity (I^2^ = 37.10%) was observed in this analysis (S5 Fig).

*Association of ICP with neonatal stillbirths*. Fifteen cohort studies reported data on the association of ICP with stillbirth. There was no significant link between ICP and a higher incidence of stillbirth (OR: 1.62, 95% confidence interval [CI]: 0.68.– 3.88, p = 0.274) with heterogeneity of (I^2^ = 0% (S6 Fig).

*Association of ICP with small for gesational age*. Our analysis of 9 cohort studies showed that the presence of ICP was not associated with infant small for gesational age (OR: 0.87, 95% confidence interval [CI]: 0.75–1.01, p = 0.070). No heterogeneity (I^2^ = 0%) was observed in this analysis (S7 Fig).

*Association of ICP with preterm birth*. Analysis of 18 cohort studies indicated that the presence of ICP was significantly associated with higher risks of preterm birth (OR: 2.64, 95% confidence interval [CI]: 1.94–3.60, p <0.001). A moderate level of heterogeneity (I^2^ = 38.54%) was observed in this analysis (S8 Fig).

## Discussion

This study aimed to summarize all existing data and assess the relationship between ICP and co-existing pregnancy complications, obstetric, and neonatal outcomes. Our findings show a significant correlation between the presence of ICP and co-existing pregnancy complications, and poorer obstetric outcomes, such as preeclampsia, higher incidence of unplanned/emergency C-sections, preterm birth, and lower gestational age. Our results also demonstrate that ICP worsens neonatal outcomes by increasing the incidence of NICU admission. However, we did not detect any substantial impact of ICP on the incidence rate of stillbirths, post-partum hemorrhage, and small for gestational age neonates.

ICP is a serious condition that can have significant negative effects on both maternal and neonatal outcomes through various mechanisms [13, 14, 38, 39]. We have organized our discussion into two distinct segments: one addressing the findings derived from the systematic review and another from the meta-analysis, offering clarity and coherence for the reader.

### Systematic review findings

Our systematic review underscores the significant correlation between the presence of ICP and co-existing pregnancy complications, which manifest in poorer obstetric outcomes. The mechanisms underlying this association are multifaceted. ICP, characterized by impaired bile flow from the liver, can lead to systemic biochemical disruptions, including elevated serum bile acid levels. These biochemical alterations may contribute to the development or exacerbation of preeclampsia, a condition characterized by endothelial dysfunction and systemic inflammation. Additionally, the cholestatic milieu in ICP may disrupt placental function, predisposing to placental ischemia and subsequent preeclampsia. Our review also revealed a heightened likelihood of emergency or unplanned C-sections in pregnancies complicated by ICP. The reasons behind this association are twofold [5, 6, 15, 36]. Firstly, the presence of ICP may necessitate expedited delivery to mitigate risks to maternal and fetal health, particularly in cases of worsening cholestasis or fetal distress [1, 5]. Secondly, maternal complications such as pruritus, fatigue, and malaise may prompt healthcare providers to opt for surgical intervention to alleviate maternal distress [4].

Moreover, our systematic review highlights the tendency for pregnancies affected by ICP to culminate prematurely, thereby amplifying the neonatal health risks associated with shorter gestational periods. The pathophysiological mechanisms driving preterm birth in ICP may include heightened inflammatory responses, impaired uteroplacental circulation, and fetal distress secondary to maternal cholestasis [1, 7, 19]. Despite an expected correlation, we did not discern substantial impacts of ICP on stillbirth rates, post-partum hemorrhage, or the incidence of small for gestational age neonates in our systematic review. The lack of significant findings in these domains may be attributed to the complex interplay of various confounding factors, including gestational age at diagnosis, severity of cholestasis, and adequacy of clinical management strategies.

### Meta-analysis findings

The meta-analysis component of our study reinforces several key observations. Firstly, it corroborates the association between ICP and adverse obstetric outcomes, particularly a heightened risk of emergency or unplanned C-sections. The pathophysiological rationale for this association lies in the potential for worsening maternal cholestasis to precipitate acute fetal distress necessitating expedited delivery via C-section [1, 7, 19]. Furthermore, our meta-analysis substantiates the increased incidence of NICU admissions among neonates born to mothers with ICP. The mechanisms underlying this phenomenon are multifaceted. Prenatal exposure to maternal cholestasis may predispose neonates to respiratory distress syndrome, transient tachypnea of the newborn, and meconium aspiration syndrome due to intrauterine hypoxia and meconium passage secondary to fetal distress [40].

Intriguingly, our meta-analysis also unveils elevated rates of maternal and neonatal morbidity in cases of severe ICP [41]. The reasons for this heightened morbidity may include the exacerbation of underlying maternal comorbidities, such as gestational diabetes or hypertension, and the increased susceptibility of neonates to respiratory and metabolic complications associated with severe cholestasis [42]. However, despite the anticipated association, our study did not yield significant findings regarding the incidence of neonatal stillbirths in pregnancies complicated by ICP. This finding underscores the complexity of stillbirth etiology, which may be influenced by multifactorial determinants beyond the scope of maternal cholestasis alone, including placental insufficiency, genetic factors, and maternal lifestyle variables.

### Limitations

Our systematic review and meta-analysis possess several limitations that warrant consideration. Firstly, the inclusion of ten retrospective cohort studies introduces the potential for bias in our results. The retrospective nature of these studies may impact the accuracy and reliability of the data, and as such, caution is advised in interpreting our findings.Secondly, a notable limitation arises from the scarcity of studies reporting neonatal outcomes, such as NICU admission and stillbirths. This paucity of data may render our meta-analysis underpowered and biased, necessitating careful interpretation of the results. The limited availability of neonatal outcome data underscores the need for more comprehensive investigations in future research.

Furthermore, our analysis reveals variability in the parameters reported across the studies included in the review. For instance, C-section rates were widely evaluated across 15 studies, while preeclampsia was only reported by seven studies. This heterogeneity in reported data introduces a potential source of variability in our analyses and may contribute to bias in our results, posing challenges to the generalizability of our findings.

A notable omission in our study is the non-inclusion of maternal total bile acids levels as a factor in our analyses. While we recognize the significance of total bile acids levels in the context of ICP, we made a deliberate choice to focus our meta-analysis on broader obstetric outcomes. This decision was made to streamline our study and maintain clarity in our investigation. However, it is important to acknowledge that the exclusion of this factor represents a limitation in our study, and future research should explore the potential role of maternal total bile acids levels in conjunction with obstetric outcomes for a more comprehensive understanding of the topic.

In conclusion, our study underscores the need for additional large-scale trials with consistent and comprehensive data reporting for both obstetric and neonatal outcomes. Replication of our findings in such studies will contribute to the development of more robust evidence, guiding clinicians in selecting appropriate prophylactic strategies for managing ICP.

### Strengths

This manuscript exhibits several strengths that contribute to its significance in the field of obstetrics and neonatology. Through a comprehensive literature review across multiple databases, including PubMed, Embase, and Web of Science, the study ensures the inclusion of recent and relevant data. Employing a robust meta-analysis methodology, the manuscript synthesizes findings from identified studies using a random-effects model, providing a quantitative estimation of the association between ICP and key pregnancy outcomes. Clear presentation of results in the manuscript enhances readability and facilitates understanding of the significant associations between ICP and adverse obstetric and neonatal outcomes such as emergency cesarean sections, preeclampsia, NICU admission, and preterm birth. The study’s clinical relevance is underscored by its potential to inform the development of comprehensive care guidelines for expectant mothers and newborns with ICP, ultimately improving patient outcomes. Furthermore, the manuscript contributes to knowledge by providing a direct evaluation of the link between ICP and neonatal obstetric outcomes, addressing a gap in the existing literature and guiding future research in this area. Notably, it represents the most comprehensive analysis of neonatal and obstetric outcomes demonstrating the influence of ICP on pregnancy, thus providing valuable insights that will inform clinicians in their decision-making processes. Overall, these strengths enhance the credibility and significance of the study, making it a valuable resource for clinicians and researchers alike.

## Conclusions

Our systematic review and meta-analysis shows a significant association of ICP with poor co-existing pregnancy complications, such as preeclampsia, and obstetric outcomes such as higher incidence of unplanned/emergency C-sections, and lower gestational age. ICP strongly correlated with worse neonatal outcomes such as NICU admission and preterm birth. There was no significant association between ICP and neonatal stillbirth, small for gestational age, and post partum hemorrhage. Our findings have significant implications for developing best practice guidelines for clinicians for improving prognostic obstetric and neonatal outcomes in mothers with ICP. By understanding the associative role between ICP and pregnancy-related outcomes, clinicians can better adapt prophylactic strategies for mothers with ICP.

## Supporting information

S1 Checklist (DOCX)

S1 Fig Unplanned/emergency C-section delivery. (TIF)

S2 Fig Incidence of preeclampsia. (TIF)

S3 Fig Incidence of postpartum hemorrhage. (TIF)

S4 Fig Lower gestational age. (TIF)

S5 Fig Incidence of NICU admission. (TIF)

S6 Fig Incidence of stillbirth. (TIF)

S7 Fig Infant small for gesational age. (TIF)

S8 Fig Incidence of preterm birth. (TIF)

S1 File (DOCX)
