# Supplementary figures and images for: Systematic review and meta-analysis: Evaluating the influence of intrahepatic cholestasis of pregnancy on obstetric and neonatal outcomes
Source: PLoS One. 2024 Jun 4;19(6):e0304604. doi: 10.1371/journal.pone.0304604 (PMC11149858; doi:10.1371/journal.pone.0304604)

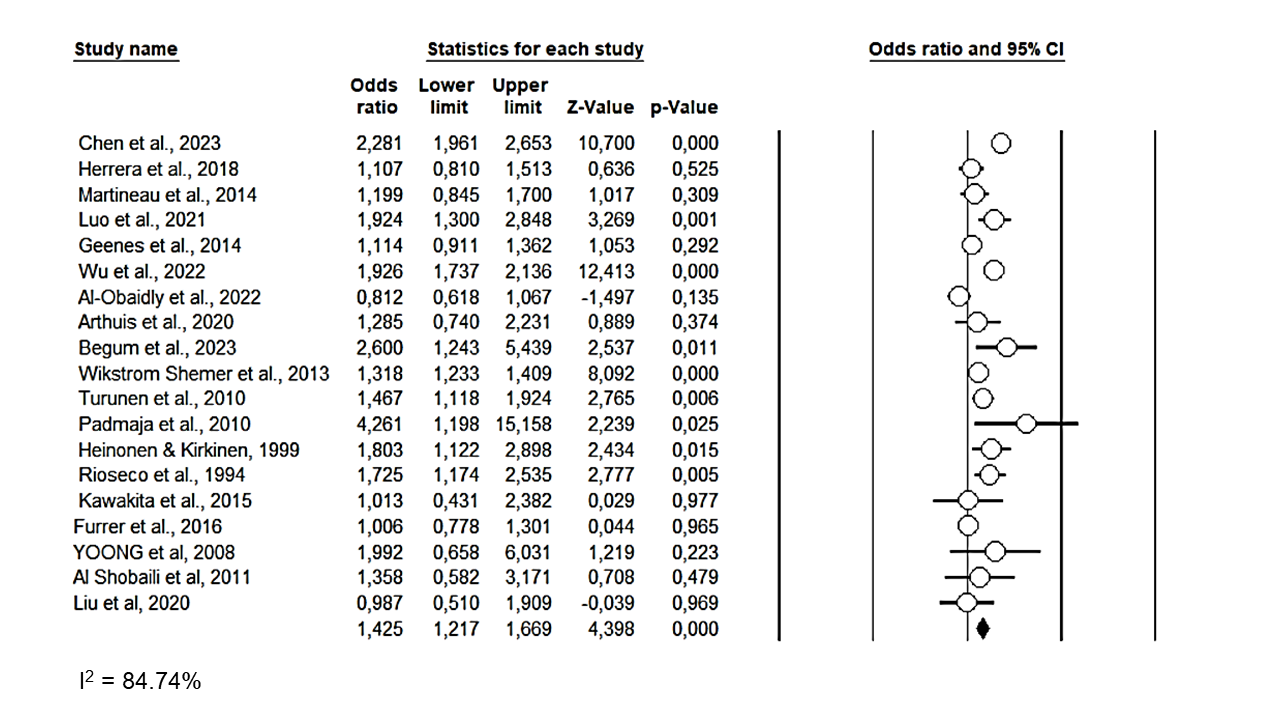

Supplement: S1 Fig — (TIF) [file pone.0304604.s002.tif]

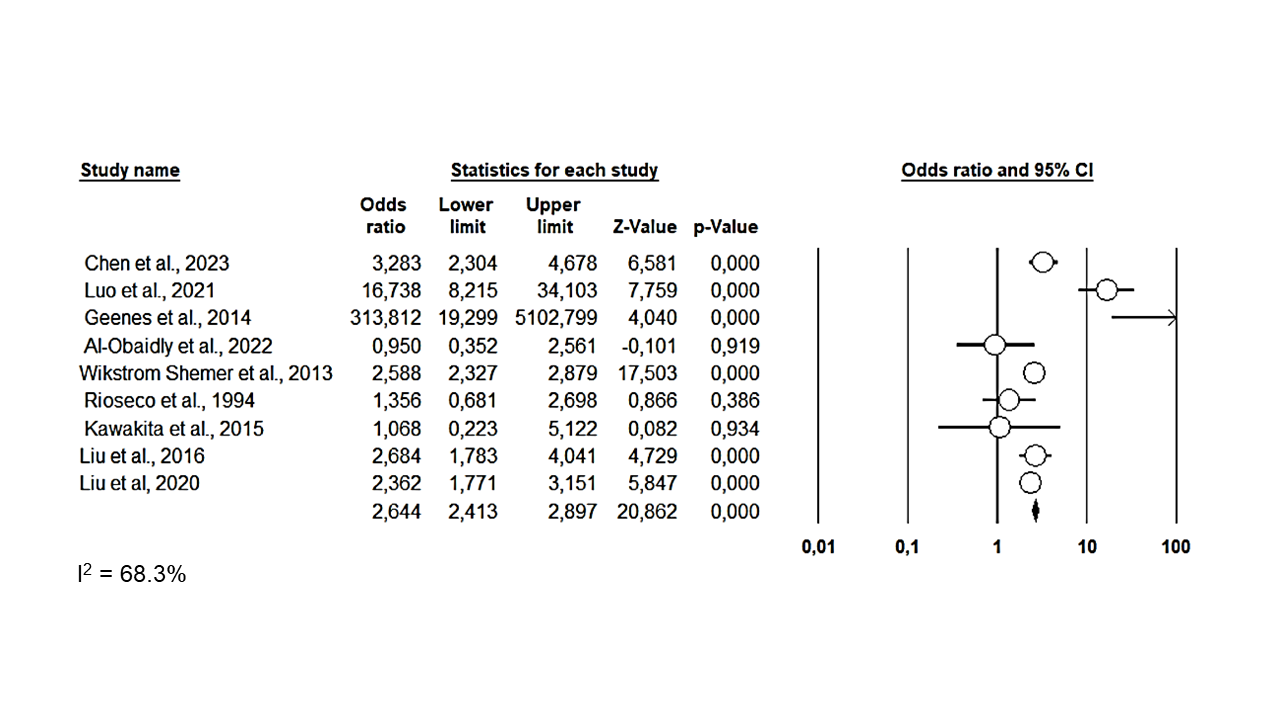

Supplement: S2 Fig — (TIF) [file pone.0304604.s003.tif]

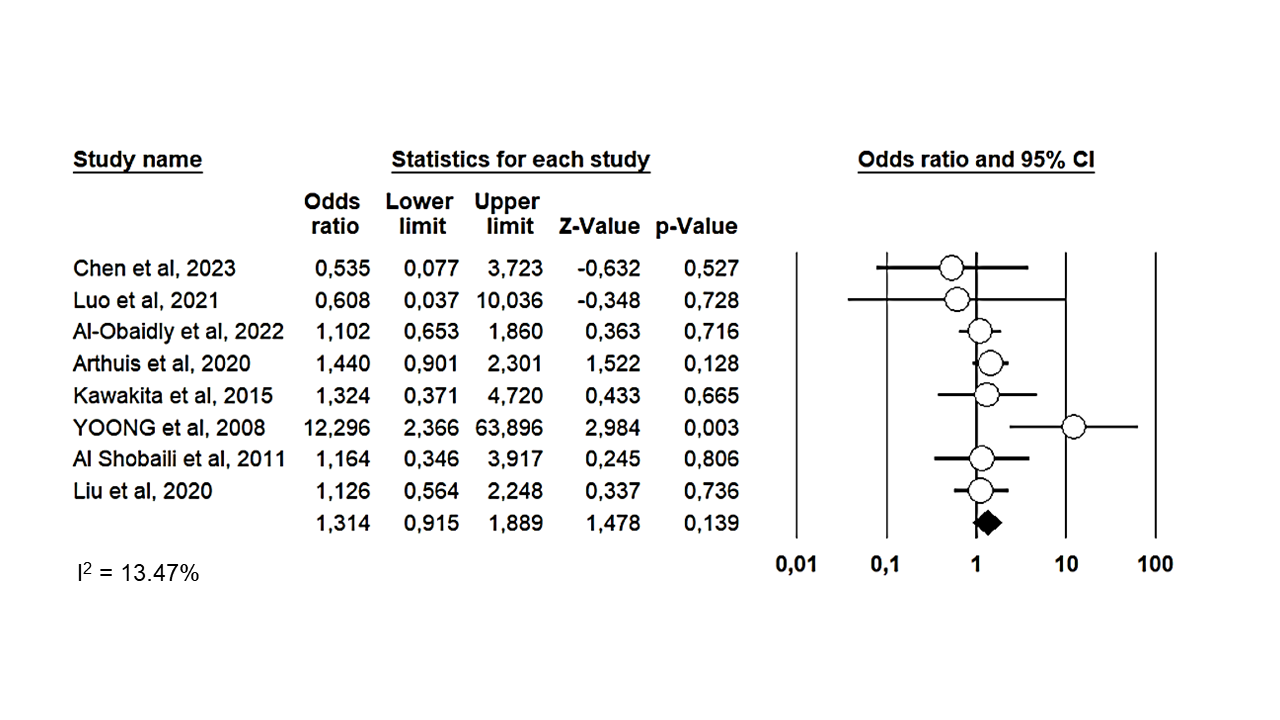

Supplement: S3 Fig — (TIF) [file pone.0304604.s004.tif]

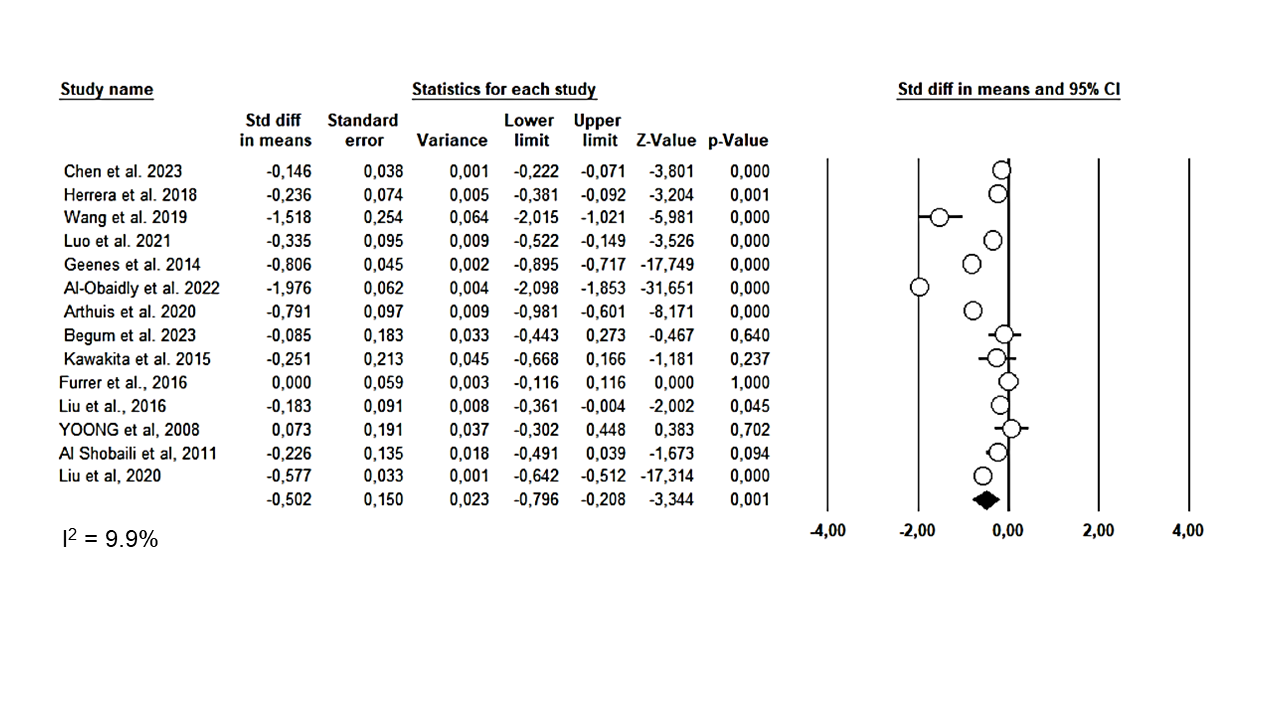

Supplement: S4 Fig — (TIF) [file pone.0304604.s005.tif]

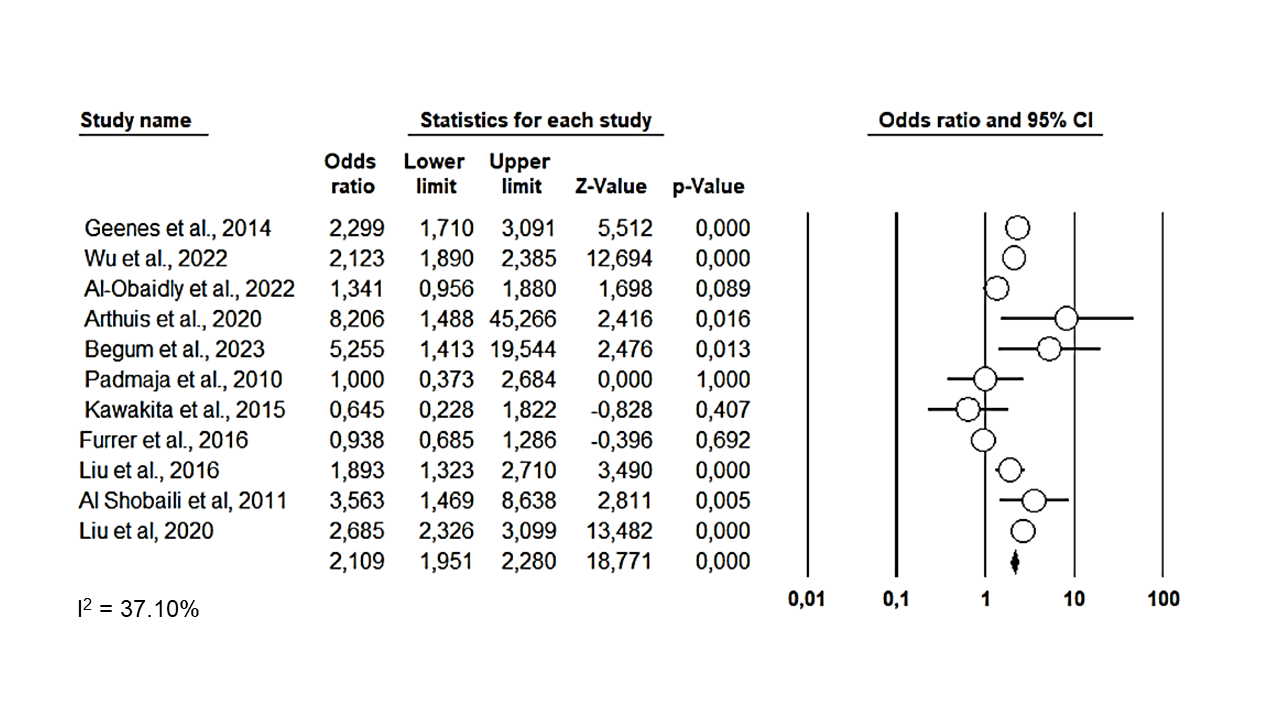

Supplement: S5 Fig — (TIF) [file pone.0304604.s006.tif]

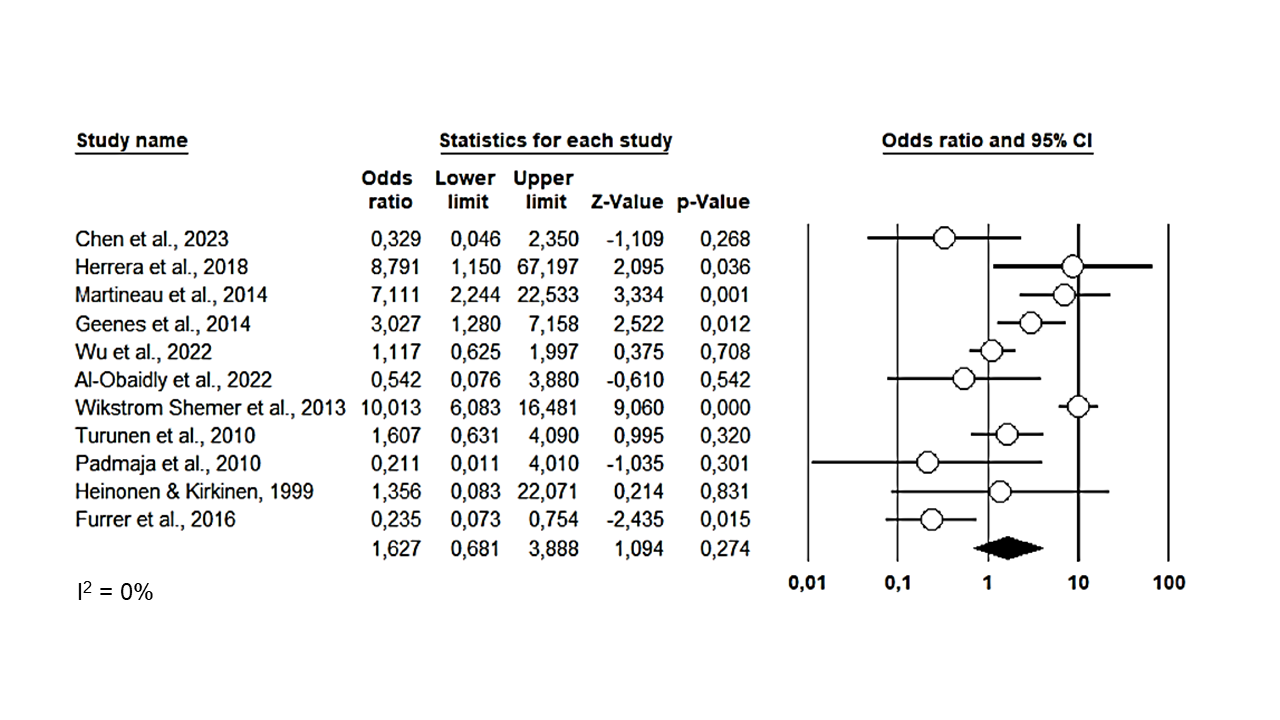

Supplement: S6 Fig — (TIF) [file pone.0304604.s007.tif]

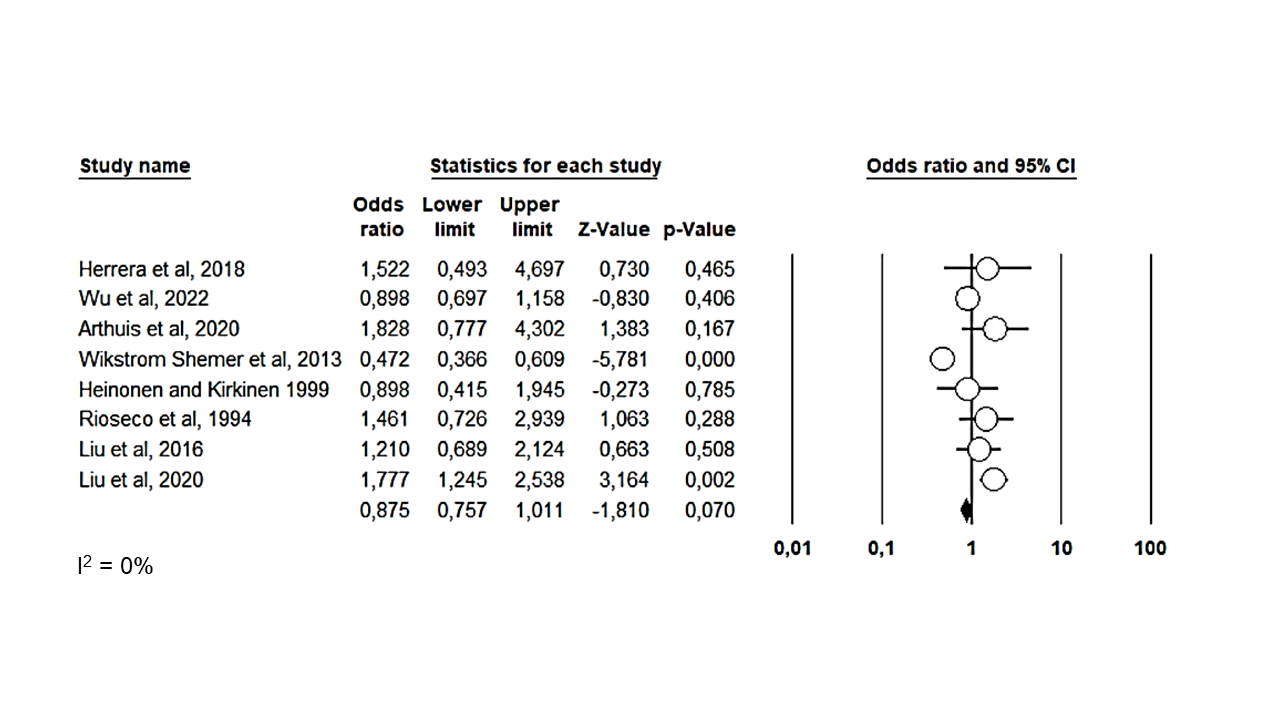

Supplement: S7 Fig — (TIF) [file pone.0304604.s008.tif]

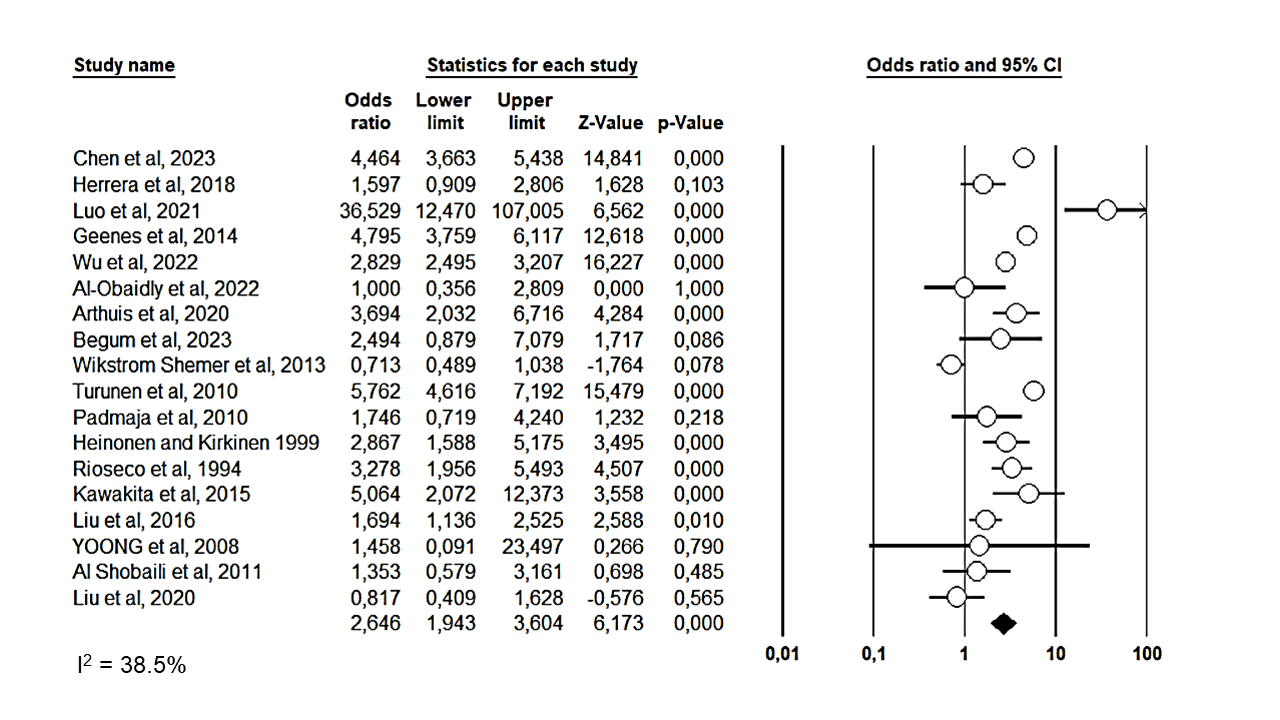

Supplement: S8 Fig — (TIF) [file pone.0304604.s009.tif]
