# Supplementary material for: Systematic review and meta-analysis: Evaluating the influence of intrahepatic cholestasis of pregnancy on obstetric and neonatal outcomes
Source: PLoS One. 2024 Jun 4;19(6):e0304604. doi: 10.1371/journal.pone.0304604 (PMC11149858; doi:10.1371/journal.pone.0304604)
Supplement: S1 File — (DOCX) [file pone.0304604.s010.docx]

Search strategy

1. Pubmed

(("Intrahepatic cholestasis of pregnancy" OR "ICP") AND ("Emergency cesarean sections" OR "Unplanned cesarean sections" OR "Cesarean delivery" OR "Preeclampsia" OR "Hemorrhage" OR "Postpartum hemorrhage" OR "Preterm birth" OR "Premature delivery" OR "Small for gestational age" OR "Stillbirth" OR "Neonatal intensive care unit" OR "NICU admission" OR "Neonatal outcomes" OR "Perinatal complications")) AND ("2000/01/01"[Date - Publication] : "2023/06/30"[Date - Publication])

1. Scopus

TITLE-ABS-KEY(("Intrahepatic cholestasis of pregnancy" OR "ICP") AND ("Emergency cesarean sections" OR "Unplanned cesarean sections" OR "Cesarean delivery" OR "Preeclampsia" OR "Hemorrhage" OR "Postpartum hemorrhage" OR "Preterm birth" OR "Premature delivery" OR "Small for gestational age" OR "Stillbirth" OR "Neonatal intensive care unit" OR "NICU admission" OR "Neonatal outcomes" OR "Perinatal complications")) AND PUBYEAR > 1999 AND PUBYEAR < 2024

1. Web of Science

TS=("Intrahepatic cholestasis of pregnancy" OR "ICP") AND TS=("Emergency cesarean sections" OR "Unplanned cesarean sections" OR "Cesarean delivery" OR "Preeclampsia" OR "Hemorrhage" OR "Postpartum hemorrhage" OR "Preterm birth" OR "Premature delivery" OR "Small for gestational age" OR "Stillbirth" OR "Neonatal intensive care unit" OR "NICU admission" OR "Neonatal outcomes" OR "Perinatal complications") AND PY=(2000-2023)
